# Cigarette brand loyalty among smokers in six European countries: Findings from the EUREST-PLUS ITC Europe Surveys

**DOI:** 10.18332/tid/99116

**Published:** 2019-02-20

**Authors:** Sarah O. Nogueira, Olena Tigova, Yolanda Castellano, Ute Mons, Christina N. Kyriakos, Ann McNeill, Antigona C. Trofor, Witold A. Zatoński, Krzysztof Przewoźniak, Tibor Demjén, Yannis Tountas, Anne C. K. Quah, Geoffrey T. Fong, Marcela Fu, Constantine I. Vardavas, Esteve Fernández, on behalf of the EUREST-PLUS consortium

**Affiliations:** 1 Catalan Institute of Oncology (ICO), Barcelona, Spain; 2 Bellvitge Biomedical Research Institute (IDIBELL), Barcelona, Spain; 3 School of Medicine and Health Sciences, University of Barcelona, Catalonia, Spain; 4 Cancer Prevention Unit and WHO Collaborating Centre for Tobacco Control, German Cancer Research Center (DKFZ), Heidelberg, Germany; 5 European Network for Smoking and Tobacco Prevention (ENSP), Brussels, Belgium; 6 University of Crete (UoC), Heraklion, Greece; 7 UK Centre for Tobacco and Alcohol Studies, King’s College London (KCL), London, United Kingdom; 8 University of Medicine and Pharmacy ‘Grigore T. Popa’ Iasi, Iasi, Romania; 9 Aer Pur Romania, Bucharest, Romania; 10 Health Promotion Foundation (HPF), Warsaw, Poland; 11 European Observatory of Health Inequalities, President Stanisław Wojciechowski State University of Applied Sciences, Kalisz, Poland; 12 Maria Skłodowska-Curie Institute-Oncology Center (MSCI), Warsaw, Poland; 13 Smoking or Health Hungarian Foundation (SHHF), Budapest, Hungary; 14 National and Kapodistrian University of Athens (UoA), Athens, Greece; 15 Department of Psychology and School of Public Health and Health Systems, University of Waterloo (UW), Waterloo, Canada; 16 Ontario Institute for Cancer Research, Toronto, Canada

**Keywords:** brand loyalty, brand choice, attachment, plain packaging, tobacco

## Abstract

**INTRODUCTION:**

This study aims to describe the degree of smokers’ loyalty to a specific brand of tobacco products and the variables related to choosing a specific brand among smokers in six European countries.

**METHODS:**

A cross-sectional analysis was conducted for a representative sample of adult smokers from Germany, Greece, Hungary, Poland, Romania, and Spain (approximately 1000 smokers per country). The prevalence of smokers’ having a usual brand of cigarettes smoked (factory-made or roll-yourown cigarettes), the brand of choice, the factors for choosing a specific brand and the degree of loyalty to that brand (not at all, a little, somewhat and a lot) were assessed by country, sociodemographics and smoking-related variables.

**RESULTS:**

In total, 86.6% of the smokers reported having a usual brand. In three out of the six countries, one brand holds the loyalty of between 17.8% and 24.5% of the smokers that reported having a usual brand for factory-made cigarettes. Most participants reported being loyal ‘a lot’ to their brand of choice (44.4%). The reasons most reported for choosing a cigarette brand were the taste (83.2%) and the price (51.7%).

**CONCLUSIONS:**

Brand loyalty is high among factory-made and roll-your-own cigarette smokers in six European countries. Future research on longitudinal trends of brand loyalty to evaluate the effect of tobacco control policies in these European countries is warranted.

## INTRODUCTION

Tobacco was the cause of the death of around seven million people in 2017^1^, with an alarming rate of up to two deaths per three smokers^2^. Although it is such a lethal product, tobacco companies still achieve high success in marketing and selling tobacco. In fact, the six largest cigarette companies made a profit of approximately $10000 per death from tobacco smoking, in 2015^3^. Understanding the factors influencing smokers’ brand choice and their loyalty to a brand are important as countries develop and implement tobacco control strategies^4^, but there is still scarce academic research in this regard.

The European Union (EU) has become an increasingly restrictive market for tobacco products in recent years with the signature of the World Health Organization (WHO) Framework Convention on Tobacco Control (FCTC)^5^, the first public health treaty aiming to tackle some of the causes of the tobacco epidemic, and the approval of a new EU Tobacco Products Directive (TPD) (2014/40/EU), which aims to regulate several aspects surrounding tobacco products^6^. Both of the abovementioned regulations could affect whether and why smokers are loyal to a cigarette brand, although none of these regulations includes measures designed to directly influence brand loyalty.

Diminishing brand loyalty might be an effective pathway to tobacco control, considering brand loyalty has been found to be associated with a decline in the sense of identity smokers may have of being a smoker of a certain brand, and therefore sharing special characteristics with those that share the same brand of choice^7,8^. Such brand identity has been used by the tobacco industry as a means to maintain sales growth. This became clear with the disclosure of tobacco industry internal documents, which have shown that tobacco companies develop explicit marketing strategies to create meaningful identities through tobacco brands^9^. Similarly, there is theoretical support for the link between brand identity and the deflection of bad connotations and stigmatization associated with smoking^10,11^. The implementation of cigarette standardised packaging design was found to be associated with decreased brand identity, decreased positive brand stereotypes, decreased smoking behaviour and higher odds of quitting among smokers^8^.

This paper documents several aspects of tobacco brand loyalty, such as the degree of brand loyalty and variables associated with brand choice of tobacco products among smokers in Germany (DE), Greece (GR), Hungary (HU), Poland (PL), Romania (RO), and Spain (ES), countries where there are still no data about such aspects of brand loyalty.

## METHODS

### Study design

The EUREST-PLUS project aims to evaluate the implementation and impact of the TPD and WHO FCTC in six European Member States (MS). The cross-sectional data analysed here derive from Wave 1 of the EUREST-PLUS International Tobacco Control Policy Evaluation (ITC) survey, a longitudinal cohort study of smokers in DE, GR, HU, PL, RO, and ES^12,13^. The fieldwork for Wave 1 was conducted between June 2016 and September 2016^14^. The survey sample comprised 6011 (about 1000 per country) nationally representative adult (over 18 years old) cigarette smokers that had smoked more than 100 cigarettes in their lives. Further details including study design and recruitment can be found elsewhere^12,13^.

### Ethics

This study’s protocol was approved by the Office of Research Ethics at the University of Waterloo (Canada), and by the ethics committees of all the participant countries and partnering institutions. The EUREST-PLUS Project is registered in Clinicaltrials. gov (registration number NCT02773836).

### Measures

#### Brand loyalty measures

Participants were asked: ‘Do you have a usual brand and variety of cigarettes?’. Response options were ‘yes’ and ‘no’. If they answered ‘yes’, they were asked the following questions to explore the degree of brand loyalty: ‘To what extent are you committed to your regular brand of cigarettes?’. Answer options were ‘not at all’, ‘a little’, ‘somewhat’, ‘a lot’, ‘don’t know’, and ‘refused’. Those that answered ‘don’t know’ and ‘refused’ were excluded from the analysis of the degree of loyalty and factors influencing the decision to choose a specific brand.

In addition, respondents were asked: ‘What is your usual brand of factory-made cigarettes?’ and ‘What is your usual brand of roll-your-own cigarettes?’ Two lists of brands of roll-your-own (RYO) and factory-made (FM) cigarettes had been previously developed for each country and the answers provided by the respondents were compared to those lists and categorized accordingly; if the brand mentioned was not in the list, the response was categorized as ‘other brand’. Participants could also respond ‘don’t know’ or ‘refused’ and those who chose one of these two options were excluded from the analysis.

#### Reasons for brand loyalty

Respondents were also asked: ‘In choosing your usual brand, was part of your decision to smoke this brand based on any of the following: It may not be bad for your health? The price? How they taste? The look and feel of the pack? The tar and nicotine levels of the brand?’. The response options for each question were ‘yes’, ‘no’, ‘don’t know’, and ‘refused’. Those who answered ‘don’t know’ or ‘refused’ were excluded from the analysis.

#### Other measures

Sociodemographic characteristics studied were country, sex (female and male), age group (18–24, 25–39, 40–54, 55 years and older), and level of education (low, medium, high). Smoking behaviours were assessed with the following variables: smoking frequency (daily, occasional), cigarettes smoked per day (≤10, 11–20, 21–30, >30), the type of cigarettes smoked (FM only, RYO only, both), and tobacco addiction, which was assessed with the Heaviness of Smoking Index (HSI; with categories low=0–2, moderate=3–4, and high=5–6)^14^.

### Analysis

Descriptive analyses were conducted to compute the prevalence of brand loyalty, the degree of loyalty (not at all, a little, somewhat, and a lot loyal), and the reasons influencing the decision for choosing their usual brand among smokers by country, sociodemographic and smoking-related variables. Pearson’s chi-squared test was used to assess differences among groups. The overall most popular brands of RYO and FM cigarettes were identified by country. Additionally, multivariate logistic regression was used to compute adjusted odds ratios (AOR) and 95% confidence intervals (CI) of the association of having a usual brand by sociodemographic variables. All the analysis incorporated the weights from the complex sampling design. Stata version 13 was used for the analyses.

## RESULTS

### Usual brand

Most respondents reported having a usual brand (86.6%). Table 1 presents the results for the differences between smokers with and without a usual brand by sociodemographic characteristics and smoking-related characteristics. GR was the country with the highest prevalence of smokers loyal to a brand (96.0%) while PL was the country with the lowest (73.9%). Females were more likely to report having a usual brand than males (88.8% vs 85.0%; AOR=1.42, 95% CI: 1.21–1.67). Smokers from the youngest age group were more likely to report having a usual brand than those who were older (87.5% of 18–24 group vs 83.2% of ≥55 years group; AOR=1.51, 95% CI: 1.06–2.17). In all, 88.6% of smokers of FM cigarettes, 85.4% of RYO cigarettes smokers and 73.0% of smokers of both types of cigarettes reported having a usual brand. The difference between those smoking both and those smoking FM cigarettes was statistically significant (AOR=2.87, 95% CI: 2.11–3.89). Of the daily and occasional smokers, 87.4% and 70.9% reported having a usual brand, respectively.

**Table 1:** Differences between smokers with and without a usual brand by country, sociodemographic variables and smoking-related variables, N=6003

	*Loyal to a brand*			*Not loyal to a brand*			AOR*	*(95% CI)*	*p*
	*n*	*%*	*95% CI*	*n*	*%*	*95% CI*			
**All**	5226	86.6	(85.2–88.0)	777	13.4	(12.0–14.8)			
**Country**									
Germany	844	81.8	(77.6–85.9)	158	18.2	(14.1–22.4)		Ref.	
Greece	956	96.0	(94.4–97.7)	43	4.0	(2.3–5.6)	5.05	(3.04–8.40)	<0.001
Hungary	918	91.0	(87.5–94.6)	82	9.0	(5.4–12.5)	2.38	(1.38–4.09)	0.002
Poland	759	73.9	(69.5–78.3)	241	26.1	(21.7–30.5)	0.41	(0.26–0.64)	<0.001
Romania	833	85.6	(82.1–89.1)	168	14.4	(10.9–17.9)	0.79	(0.49–1.26)	0.322
Spain	916	91.3	(88.7–93.9)	85	8.7	(6.1–11.3)	1.99	(1.24–3.20)	0.005
**Sex**									
Male	2718	85.0	(83.2–86.7)	454	15.0	(13.3–16.8)		Ref.	
Female	2508	88.8	(87.4–90.3)	323	11.2	(9.7–12.6)	1.42	(1.21–1.67)	<0.001
**Age (years)**									
18–24	439	87.5	(84.4–90.6)	67	12.5	(9.4–15.6)	1.51	(1.06–2.17)	0.024
25–39	1557	88.1	(85.9–90.3)	212	11.9	(9.7–14.1)	1.47	(1.14–1.90)	0.003
40–54	1763	87.4	(85.7–89.2)	240	12.6	(10.8–14.3)	1.41	(1.14–1.75)	0.002
≥55	1467	83.2	(80.7–85.7)	258	16.8	(14.3–19.3)		Ref.	
**Level of education**									
Low	1931	87.4	(85.4–89.4)	278	12.6	(10.6–14.6)		Ref.	
Medium	2668	85.3	(83.5–87.1)	434	14.7	(12.9–16.5)	1.12	(0.90–1.40)	0.296
High	596	90.2	(87.4–93.0)	60	9.8	(7.0–12.6)	1.51	(0.98–2.32)	0.060
**Type of cigarettes smoked**									
Factory-made	3944	88.6	(87.1–90.1)	510	11.4	(9.9–12.9)	2.87	(2.11–3.89)	<0.001
Roll-your-own	916	85.4	(81.7–89.1)	144	14.6	(10.9–18.3)	1.10	(0.77–1.56)	0.598
Both	366	73.0	(68.6–77.4)	122	27.0	(22.6–31.4)		Ref.	
**Frequency of smoking**									
Daily	5015	87.4	(86.0–88.8)	696	12.6	(11.2–14.0)			
Occasional	211	70.9	(65.0–76.9)	81	29.1	(23.1–35.0)			
**Cigarettes smoked/day**									
≤ 10	1795	86.2	(84.3–88.1)	288	13.8	(11.9–15.7)			
11–20	2699	87.4	(85.7–89.2)	375	12.6	(10.8–14.3)			
21–30	453	84.1	(80.0–88.3)	70	15.9	(11.7–20.0)			
> 30	274	85.7	(81.3–90.1)	40	14.3	(9.9–18.7)			
Tobacco addiction**									
Low	2077	88.0	(86.1–89.8)	280	12.0	(10.2–13.9)	1.31	(0.94–1.81)	0.113
Moderate	2480	87.7	(85.9–89.4)	332	12.3	(10.6–14.1)	1.57	(1.17–2.11)	0.002
High	440	83.5	(79.8–87.3)	77	16.5	(12.7–20.2)		Ref.	

AOR: adjusted odds ratio, CI: confidence interval.

* Adjusted odds ratios derived from multi-level logistic regression, adjusted for age, sex, educational level, area of residence, and tobacco addiction.

** Measured with the Heaviness of Smoking Index: 0–2=low; 3–4=moderate; 5–6=high.

### Brands most used across countries

The most popular brand in the overall sample was Malboro (14.65%). In three out of the six countries, Marlboro was the usual brand for between 17.8% and 24.5% of smokers that reported having a usual brand for FM cigarettes, and it was the most popular in four of the six countries. The second most popular brand, Kent, reaches this position because of RO smokers (23.3%), but was not highly reported as the brand of choice in the other five countries. RO participants were the ones reporting the most disperse results, with 76.7% of the smokers being loyal to brands other than the most popular ones in the overall sample. Brand loyalty for RYO was more dispersed, with Marlboro being the only usual brand for more than 10%, in two out of the six countries.

### Degree of loyalty to usual brand

Table 2 presents the self-reported smokers’ degree of loyalty to their usual brand by country, sociodemographics and tobacco addiction. The degree of loyalty to a brand was significantly different within all the studied variables (p<0.001), except for sex (p=0.413). Most participants reported being ‘a lot’ loyal to their brand of choice (44.4%), 34.3% reported being ‘somewhat’ loyal, 16.2% ‘a little’, and 5.1% ‘not at all’ loyal. Participants in GR (51.5%), HU (52.1%), and RO (64%) were the ones with the highest levels of loyalty to a brand, while those in DE had a homogeneous distribution across the four categories of brand loyalty. A gradient in the degree of brand loyalty was observed in participants across sexes, age groups, and levels of education. Participants across these three sociodemographic variables were between 3.3% and 6.8% ‘not at all’ loyal, between 14.0% and 19.3% ‘a little’ loyal, between 32.4% and 38.3% ‘somewhat’ loyal, and between 35.6% and 46.2% ‘a lot’ loyal to their brand of choice (p<0.001). Another noticeable gradient was observed among those reporting being ‘a lot’ loyal to a brand, varying from 41.4% for those less addicted to 56.0% of those highly addicted to nicotine (p<0.001).

**Table 2:** Degree of loyalty to the brand by country, sociodemographic variables and tobacco addiction, N=5196

	*Not at all*			*A little*			*Somewhat*			*A lot*		
	*n*	*%*	*95% CI*	*n*	*%*	*95% CI*	*n*	*%*	*95% CI*	*n*	*%*	*95% CI*
**All**	276	5.1	(4.3–5.9)	852	16.2	(14.8–17.5)	1760	34.3	(32.3–36.3)	2308	44.4	(42.2–46.7)
**Country**												
Germany	169	19.3	(15.3–23.3)	238	28.9	(25.1–32.7)	226	27.3	(23.4–31.2)	204	24.5	(19.3–29.7)
Greece	3	0.3	(0.0–0.5)	45	4.3	(3.0–5.7)	385	43.9	(38.3–49.6)	523	51.5	(46.1–56.8)
Hungary	13	1.1	(0.4–1.7)	173	19.1	(15.0–23.4)	258	27.7	(23.8–31.5)	472	52.1	(46.2–58.0)
Poland	24	3.0	(1.5–4.6)	177	23.7	(19.7–27.6)	301	39.2	(35.2–43.3)	240	34.1	(30.1–38.1)
Romania	23	3.0	(1.5–4.4)	69	9.2	(6.4–12.0)	205	23.8	(19.5–28.1)	533	64.0	(58.6–69.4)
Spain	44	5.1	(3.5–6.8)	150	14.8	(11.5–17.9)	385	42.9	(37.8–48.1)	336	37.2	(32.0–42.5)
p-value			<0.001									
**Sex**												
Male	149	5.7	(4.7–6.7)	423	15.7	(13.9–17.4)	917	34.5	(32.3–36.9)	1218	44.1	(41.5–46.6)
Female	127	4.3	(3.5–5.2)	429	16.8	(15.0–18.6)	843	33.9	(31.3–36.5)	1090	45.0	(42.1–47.8)
p-value			0.413									
**Age (years)**												
18–24	35	6.8	(4.2–9.3)	86	19.3	(14.7–23.9)	174	38.3	(33.6–43.0)	142	35.6	(30.8–40.5)
25–39	75	4.0	(3.0–5.1)	289	17.6	(15.3–19.9)	522	33.7	(30.9–36.4)	663	44.7	(41.4–48.0)
40–54	96	5.6	(4.4–6.8)	256	14.0	(12.1–15.9)	584	34.2	(31.2–37.2)	812	46.2	(42.7–49.6)
≥55	70	5.0	(3.7–6.3)	221	16.0	(13.8–18.1)	480	33.7	(30.5–36.8)	691	45.3	(41.9–48.8)
p-value			<0.001									
**Level of education**												
Low	115	5.9	(4.6–7.1)	353	17.8	(15.6–19.9)	623	32.4	(29.4–35.5)	838	43.9	(40.5–47.4)
Medium	137	4.8	(3.8–5.9)	411	15.5	(13.8–17.1)	919	35.3	(32.7–37.8)	1177	44.4	(41.8–47.1)
High	20	3.3	(1.8–4.8)	84	14.3	(11.1–17.4)	210	36.3	(31.8–40.9)	279	46.1	(41.5–50.8)
p-value			<0.001									
Tobacco addiction*												
Low	115	5.1	(4.1–6.1)	367	17.7	(15.7–19.7)	723	35.8	(33.4–38.2)	860	41.4	(38.7–44.1)
Moderate	109	4.5	(3.5–5.6)	390	15.4	(13.5–17.2)	822	33.8	(31.0–36.6)	1145	46.3	(43.1–49.5)
High	28	6.1	(3.3–8.9)	36	7.9	(5.4–10.4)	133	30.0	(24.8–35.1)	241	56.0	(50.9–61.2)
p-value			<0.001									

CI: confidence interval.

* Measured with the Heaviness of Smoking Index: 0–2=low; 3–4=moderate; 5–6=high

### Reasons for choosing usual brand

Table 3 presents the reasons that may influence the decision for loyalty to a specific brand by sociodemographic variables. Overall, the taste from the cigarette was the reason most cited by smokers (83.2%) followed by the price (51.7%), the tar and nicotine levels of the brand (41.5%), the look and the feel of the pack (23.2%), and the perception of being less harmful (21.4%).

Female smokers were significantly more influenced than male smokers by the price (53.2% vs 50.5%), the tar and nicotine levels (44.5% vs 39.2%), the look and feel of the pack (25.5% vs 21.4%), and the cigarette harm perception (23.8% vs 19.6%). The younger the smokers the bigger the percentage of them reporting the look and the feel of the pack as relevant (28.1% of those between 18–24 years of age vs 20.8% of those ≥55 years). In contrast, the older the age group of a smoker the bigger the percentage reporting that they considered tar and nicotine levels in choosing a brand (43.3% of those ≥55 years vs 37.1% of those between 18–24 years). These differences by age groups were statistically significant.

**Table 3:** Factors that may influence the decision to choose a specific brand by country and sociodemographic variables, N=6003

	*The taste*			*The price*			*The tar and nicotine levels of the brand*			*The look and feel of the pack*			*It may not be as bad for the health*		
	*n*	*%*	*95% CI*	*n*	*%*	*95% CI*	*n*	*%*	*95% CI*	*n*	*%*	*95% CI*	*n*	*%*	*95% CI*
**All**	4314	83.2	(81.7–84.7)	2666	51.7	(49.8–53.5)	2104	41.5	(39.3–43.8)	1190	23.2	(21.3–25.1)	1092	21.4	(19.5–23.4)
**Country**															
Germany	746	89.6	(86.9–92.2)	370	45.3	(40.1–50.4)	305	36.2	(31.4–41.1)	155	18.7	(14.7–22.7)	116	13.8	(10.6–17.0)
Greece	858	90.4	(87.8–93.0)	434	48.6	(43.6–53.5)	583	59.5	(52.9–66.2)	295	30.2	(24.8–35.7)	158	18.2	(12.1–24.3)
Hungary	707	78.4	(73.5–83.4)	628	69.1	(64.7–73.6)	340	38.2	(32.5–43.9)	201	21.8	(17.3–26.3)	252	28.0	(23.4–32.6)
Poland	644	84.9	(81.2–88.7)	502	66.7	(61.9–71.5)	283	39.7	(33.9–45.6)	199	26.4	(22.5–30.2)	177	23.1	(18.5–27.6)
Romania	606	73.1	(68.5–77.6)	327	38.7	(34.5–42.9)	442	57.4	(52.9–61.9)	256	33.8	(28.6–39.0)	283	33.3	(29.0–37.6)
Spain	753	83.0	(79.5–86.5)	405	43.3	(38.5–48.1)	151	17.4	(12.3–22.5)	84	8.9	(5.3–12.5)	106	13.2	(8.0–18.4)
p–value			<0.001			<0.001			<0.001			<0.001			<0.001
**Sex**															
Male	2252	83.8	(81.9–85.6)	1328	50.5	(48.3–52.8)	1038	39.2	(36.5–41.9)	573	21.4	(19.2–23.5)	526	19.6	(17.5–21.7)
Female	2062	82.6	(80.7–84.4)	1338	53.2	(50.7–55.6)	1066	44.5	(41.9–47.2)	617	25.5	(23.1–27.9)	566	23.8	(21.3–26.3)
p–value			0.579			<0.001			<0.001			<0.001			<0.001
**Age (years)**															
18–24	354	80.9	(76.3–85.5)	208	46.1	(40.1–52.1)	153	37.1	(31.6–42.5)	123	28.1	(23.3–32.9)	101	23.9	(19.5–28.4)
25–39	1304	82.9	(80.6–85.3)	794	52.0	(49.2–54.9)	620	42.1	(39.0–45.2)	403	27.3	(24.2–30.3)	302	21.1	(18.4–23.9)
40–54	1456	83.3	(81.3–85.3)	894	51.3	(48.5–54.1)	743	41.2	(38.1–44.2)	357	19.5	(16.9–22.1)	371	20.5	(17.6–23.3)
≥55	1200	84.5	(82.4–86.7)	770	54.1	(51.0–57.2)	588	43.3	(39.6–46.9)	307	20.8	(18.4–23.2)	318	22.1	(19.5–24.8)
p–value			0.340			0.230			0.045			<0.001			0.236
**Level of education**															
Low	1570	81.7	(79.3–84.2)	1078	55.6	(52.8–58.3)	678	36.0	(32.6–39.3)	389	19.5	(16.9–22.2)	379	20.4	(17.7–23.1)
Medium	2214	83.9	(82.2–85.5)	1339	50.8	(48.2–53.4)	1119	43.7	(40.8–46.6)	640	24.9	(22.6–27.2)	565	21.6	(19.5–23.8)
High	507	85.4	(82.2–88.7)	234	42.4	(37.2–47.6)	292	49.8	(45.4–54.3)	153	27.2	(23.0–31.3)	140	23.9	(19.6–28.1)
p–value			0.083			<0.001			<0.001			<0.001			0.086
Tobacco addiction*															
Low	1736	84.3	(82.2–86.3)	994	49.2	(46.4–52.1)	844	41.5	(38.7–44.4)	479	23.8	(21.4–26.2)	475	23.7	(21.1–26.3)
Medium	2024	82.4	(80.4–84.4)	1375	55.3	(53.1–57.6)	996	41.6	(38.5–44.6)	546	22.3	(20.0–24.5)	493	20.3	(17.9–22.6)
High	356	81.4	(77.5–85.3)	218	49.2	(44.2–54.3)	178	41.5	(36.1–46.9)	94	22.1	(16.7–27.6)	59	14.3	(10.5–18.2)
p-value			0.261			<0.001			0.969			0.623			<0.001

CI: confidence interval.

* Measured with the Heaviness of Smoking Index: 0–2=now; 3–4=moderate; 5–6=high.

## DISCUSSION

Overall, most smokers (86.6%) reported having a usual brand, particularly daily smokers (87.4%) and those highly educated (90.2%). One brand was the most popular in four out of the six countries, holding the loyalty of less than a quarter of the smokers in these countries. Almost half of the smokers reported being loyal ‘a lot’ to a brand and only 5.1% reported having the lowest level of loyalty. Taste was reported by eight in ten as a factor that influences their loyalty, and five in ten mentioned price as an influential factor.

Smokers with the lowest educational level were less brand loyal and reported price as the main influential factor. Other studies have found similar results, with smokers with lower socioeconomical levels switching brands more frequently because of price differences, i.e. being less brand loyal^15,16^. One of these studies also found similar results in relation to level of nicotine addiction, with those more addicted to nicotine being more brand loyal than those less addicted^16^. One possible explanation for these results is that those less addicted are mostly occasional smokers and tend to share cigarette packs with other smokers making their choice more defined by the others.

Gender differences were observed in reporting brand loyalty and factors influencing it, with females being more frequently loyal to a brand and mentioning price, the levels of nicotine and tar, the look and feel of the pack, and the perceived potential harms as factors influencing their choice of a cigarette brand more than males. These results might be related to the fact that in the EU, in 2016, the gender pay gap (the average difference between the renumeration for men and women working) was 16.2% and all the countries in the study the gender pay gap was at least 15.3%, except in RO (5%)^17^. Evidence also presents women as more attentive to health-related issues and more likely to take action on these matters^18–20^. Additional evidence related to brand loyalty and gender differences, that might at least partially explain our results, is that the tobacco industry has systematically targeted women in their advertisements, associating brands with positive images of independence, self-care and success^16,21,22^.

Marlboro, the most popular choice of usual brand overall, was also the most popular choice for RYO and FM smokers in four out of the six countries. Marlboro was the most sold cigarette brand in 2016 in DE, GR, HU, and ES; with DE being the country with the most affordable Marlboro cigarettes, followed by ES, GR, and HU^23–26^. As the price was the second most cited reason for choosing a brand (51.7% of the sample), the increase in taxation of cigarettes could play a part in the decrease of brand loyalty in these countries.

A difference of approximately 20% prevalence was observed among the countries with the highest and lowest brand loyalty prevalence. This finding might point to cross-country differences in the implementation of tobacco control policies, such as bans on tobacco advertising. The EUREST-PLUS ITC 6E Survey shows that tobacco advertising, promotion and sponsorship tended to be noticed more often in countries with less restrictive regulation (e.g. DE and GR)^27^. Another finding from the same EURESTPLUS ITC 6E Survey is that tobacco advertising exposure was widely prevalent outside and inside the points-of-sale in all six countries, including HU and RO, countries where bans on this advertising have been implemented^27^.

Taste of the cigarette was the factor most cited as a reason for choosing a brand by 8 in 10 smokers, followed by price (approximately 5 in 10 smokers), tar and nicotine levels for brand (4 in 10 smokers), look and the feel of the pack (2 in 10 smokers), and perception of harms (2 in 10 smokers). Data from the Eurobarometer also indicated that just under half of the smokers in the EU smoked cigarettes with special characteristics in 2017^28^. The most popular choices were the additive-free or organic cigarettes (17%) and the light cigarettes (16%). Our data point to the possibility that most smokers in Europe smoke the cigarettes they perceive as less harmful, even though there is no evidence for differences in the safety of combustible cigarettes^29,30^. Additionally, menthol flavour (8%) and other flavours (7%) were also identified in the Eurobarometer survey as consistently used by smokers. Therefore, these results reinforce the importance of the effective ban of flavourings in cigarettes established by the TPD, which should be fully implemented by 2020^6^.

Another measure that has been found to be related to brand loyalty is plain packaging^8^. It aims to standardise and eliminate the design and packaging characteristics that could mislead consumers, suggesting benefits in terms of less harm. Several studies provide evidence that it may reduce false beliefs that certain brands are less harmful and reduce pack and product appeal^9,31,32^. At the population level, plain packaging has already been proven to be effective in tackling tobacco consumption by lowering brand loyalty in Australia^8^. Additionally, Australian smokers were more likely to find their tobacco product packs less attractive, find their cigarettes less satisfying, and consider cigarette brands not differing in prestige, after one year of plain packaging implementation^8^.

In the EU, most of the measures transposed and implemented were not designed to undermine the relationship of smokers with their specific brand, which could act as a shield against tobacco cessation^9^. Meanwhile, measures such as plain packaging, that could lead to a direct change in brand loyalty, are not compulsory and were left in charge of each country by the TPD^33^. This may result in delays and interference of the tobacco industry in the process of adopting plain packaging^33^.

Within the participating countries of the EUREST-PLUS Project, HU is going to implement plain packaging in 2018, which will enable a cross-country comparison of the effects of such a measure in a longitudinal analysis. Longitudinal analysis of the EUREST-PLUS ITC Europe Survey will provide a clearer assessment of the potential association between the plain packaging policies implemented in some of the participating countries and changes in brand loyalty. Longitudinal analysis will also allow us to explore associations between brand loyalty and smoking cessation outcomes.

This study has some limitations, which should be noted. The pre-specified answer options for the questions might potentially exclude some important elements that influence brand choice and loyalty among smokers. The data used in this study are cross-sectional and this design precludes any inference about the direction of causality. The question evaluating the reasons that influence a smokers’ brand choice could be interpreted in two ways: 1) smokers choose their cigarettes because of their higher content of nicotine and tar, and 2) smokers choose their cigarettes because of their lower content of nicotine and tar as a healthier option. Although it seems intuitive they would choose the cigarettes with less concentrations of these compounds, the question could have been clearer. Some strengths should also be noted. This study used nationally representative samples of smokers in each of the six countries, making the generalisation of these results more feasible. Also, a standardised questionnaire and methodology were used for all the countries included in the current study, thus assuring comparability across countries.

## CONCLUSIONS

This study provides a snapshot of cigarette brand loyalty across six European countries for the first time. The measures used here suggest that brand loyalty is high among smokers in these EU countries.

## CONFLICTS OF INTEREST

The authors declare that they have no competing interests, financial or otherwise, related to the current work. K. Przewoźniak reports grants and personal fees from the Polish League Against Cancer, outside the submitted work. C. I. Vardavas reports that he is the Strategic Development Editor of TID and that there are no conflicts of interest with this current work. The rest of the authors have also completed and submitted an ICMJE form for disclosure of potential conflicts of interest.
